# Prevalence of emphysema in people living with human immunodeficiency virus in the current combined antiretroviral therapy era: A systematic review

**DOI:** 10.3389/fmed.2022.897773

**Published:** 2022-09-21

**Authors:** Hedda Ringheim, Rebekka F. Thudium, Jens-Ulrik S. Jensen, Omid Rezahosseini, Susanne D. Nielsen

**Affiliations:** ^1^Viro-Immunology Research Unit, Department of Infectious Diseases 8632, Rigshospitalet, University of Copenhagen, Copenhagen, Denmark; ^2^Section of Respiratory Medicine, Department of Medicine, Herlev and Gentofte Hospital, University of Copenhagen, Hellerup, Denmark; ^3^Department of Clinical Medicine, Faculty of Health and Medical Sciences, University of Copenhagen, Copenhagen, Denmark

**Keywords:** HIV, emphysema, comorbidity, systematic review, antiretroviral therapy

## Abstract

Before introducing combination antiretroviral therapy (cART), a higher prevalence of emphysema in people living with HIV (PLWH) than in the background population was reported. This systematic literature review aimed to investigate the prevalence of emphysema in PLWH and to compare the prevalence between PLWH and controls in the current cART era. A systematic literature search was conducted in PubMed, EMBASE, Scopus, and Web of Science (WOS), searching for “human immunodeficiency virus (HIV)” and “emphysema” from January 1, 2000 to March 10, 2021. Eligible studies were published after the introduction of cART, included PLWH, and reported the prevalence of emphysema. A total of 17 studies were included, and nine studies also included controls. The weighted average prevalence of emphysema in PLWH was 23% (95% CI: 16–30). In studies including both PLWH and controls the weighted average prevalence were 22% (95% CI: 10–33) and 9.7% (95% CI: 2.3–17), respectively (*p* = 0.052). The prevalence of emphysema in never-smoking PLWH and controls was just reported in one study and was 18 and 4%, respectively (*p* < 0.01). Thirteen of the studies had a moderate risk of bias, mainly due to selection of patients. A tendency to higher prevalence of emphysema was found in PLWH in comparison to controls in the current cART era. However, in the included studies, the definition of emphysema varied largely. Thus, to have a clear overview of the prevalence, further studies with well-designed cohorts of PLWH and controls are warranted.

## Introduction

During the early years of the human immunodeficiency virus (HIV) epidemic, acquired immunodeficiency syndrome (AIDS)-related emphysema was reported among people living with HIV (PLWH) (1). Emphysema is a chronic lung disease that is characterized by destruction of alveoli, and smoking, exposure to environmental pollutants, aging, infections, and conditions such as alpha-1-antitrypsin deficiency are among the risk factors (2, 3). The prevalence estimations for emphysema vary in different age groups and by different diagnostic cut-offs, ranging from 0.6 to 24% in the background population (4–7). Before introducing combined antiretroviral therapy (cART), studies reported that both smoking and non-smoking PLWH were more susceptible to emphysema than the background population (8, 9). However, it is uncertain whether there is still an increased prevalence among PLWH after introducing ART. We aimed to investigate the prevalence of emphysema in PLWH and to compare the prevalence between PLWH and controls in the current cART era.

## Materials and methods

### Search strategy and selection criteria

**FIGURE 1:**
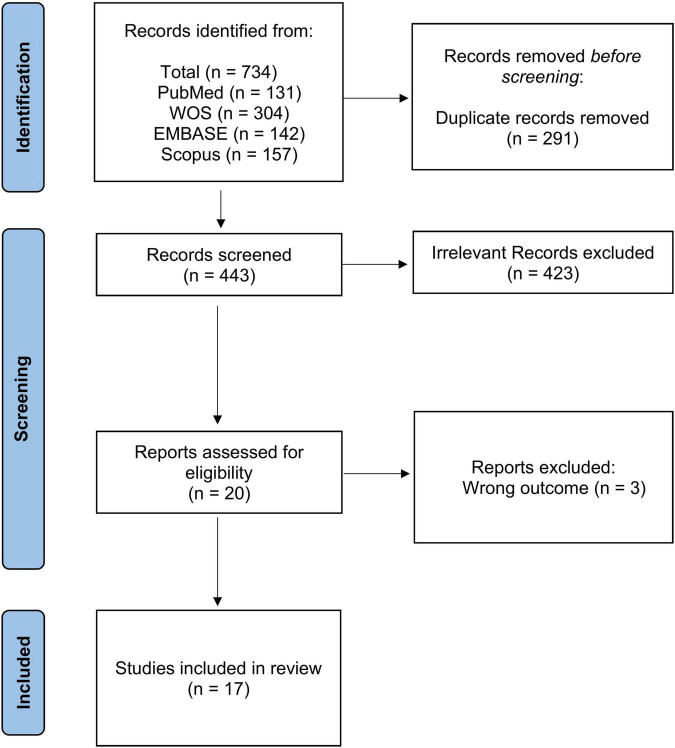
Preferred reporting items for systematic reviews and meta-analyses (PRISMA) flow chart. Legend to figure: PRISMA flow diagram for the included studies showing the selection process.

We used the Preferred Reporting Items for Systematic Reviews and Meta-Analyses (PRISMA) statement (10). The clinical question was: What is the prevalence of emphysema in PLWH and non-HIV controls in the current cART era? The clinical question was designed according to the PICOS process (10). The systematic literature review was performed in PubMed, EMBASE, Scopus and Web of Science (WOS) from January 1, 2000 to March 10, 2021. Using the same combination of keywords, we did a complementary search in google. We also read the references list of the retrieved papers for any relevant articles (Figure 1). Two investigators separately performed the searches and screened the retrieved papers by title and abstract. The relevant papers were read in full text and included if they met the inclusion criteria. A third investigator resolved potential conflicts.

### Full electronic search strategy in PubMed

We searched PubMed using MeSH terms from January 1, 2000 to March 10, 2021 and got 15 results. The MeSH terms were: ((“HIV”[Mesh]) OR “AIDS”[Mesh]) AND (“Pulmonary Emphysema”[Mesh] OR “Emphysema”[Mesh]).

In addition, a free-text search was performed using the following terms: (((hiv) OR (aids)) OR (AIDS)) AND ((emphysema) OR (pulmonary emphysema)). This search yielded 116 results. All the search results were imported into Covidence for screening (10).

### Eligibility criteria

We did not include studies that were published prior to 2000, to ensure participants were receiving modern cART. All duplicates, as well as all non-English studies and studies on non-human participants were excluded. Further, we excluded studies that diagnosed emphysema clinically or according to spirometry but without doing computed tomography (CT) scans. We included randomized controlled trials, cohort studies, case-control studies, and cross-sectional studies. Systematic reviews and meta-analyses, case reports, and expert opinions/editorials were considered ineligible to ensure stronger level of scientific evidence.

### Data extraction

**FIGURE 2:**
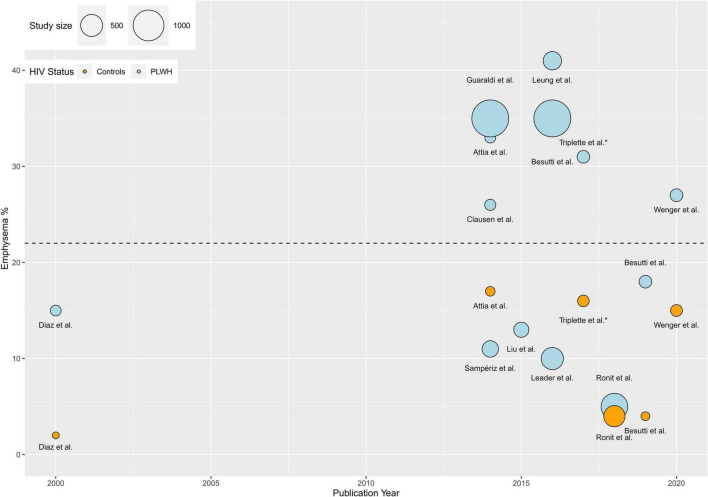
Visual representation of prevalence in PLWH and controls. A visual presentation of the prevalence of emphysema in all the included studies is presented. The weighted average prevalence of emphysema was 23% (95% CI: 16–30) (Horizontal black dotted-line). Triplette et al. published four studies on emphysema prevalence with similar results (prevalence = 31–33% in all studies). Only one of the four studies is shown in the plot for simplicity. The study by Maitre et al. (7) was register-based and included 10,067 hospitalized PLWH and 8,244,682 hospitalized non-HIV controls, and it did not include a definition of emphysema. Therefore, the study by Maitre et al. (7) differed substantially from the others in its study design, population size, and estimations. Lack of emphysema definition prohibits comparison with other studies. Furthermore, including this study in the analyses would cause bias in estimations, and we chose to exclude this study from the calculations.

We extracted data using the Extraction 2.0 in Covidence (10). The retrieved studies were thoroughly reviewed, devoting particular attention to their methods and main findings. Specific attention was brought to how each study assessed emphysema as an outcome. The prevalence of emphysema in each study was determined and visually represented with the statistical program R (Figure 2). The weighted average prevalence of emphysema in PLWH and controls was calculated as: prevalence (%) × (N/the sum of all N). The study by Maitre et al. (7) was register-based and included 10,067 hospitalized PLWH and 8,244,682 hospitalized non-HIV controls, and it did not include a definition of emphysema. Therefore, the study by Maitre et al. (7) differed substantially from the others in its study design, population size, and estimations. Lack of emphysema definition prohibits comparison with other studies. Furthermore, including this study in the analyses would cause bias in estimations, and we chose to exclude this study from the calculations. Furthermore, Triplette et al. published four studies with similar prevalences (5, 6, 11, 12). Therefore, average number of PLWH and controls by Triplette et al. was included in the calculations. Unpaired two-samples *T*-test was used to compare the weighted average prevalence and a *p* ≤ 0.05 was considered statistically significant. In a sensitivity analysis and to find the effect of new cART drugs on the prevalence of emphysema, we included studies published in 2016 and later (13).

### Risk of bias assessment of included studies

All the included studies were non-randomized, and we used Cochrane risk of bias tool for non-randomized studies (ROBINS-I) (14). We utilized the visualization tool, robvis, to produce weighted bar plots and traffic light plots (15). ROBIN-I contains seven domains with signaling questions that provide a structured approach to the risk of bias (14). The traffic light plots show a low, moderate, or high risk of bias within these important domains and also imply an overall risk of bias for each study. Additionally, the weighted bar plots depict a summary of the judgment within each domain and the studies in general.

Due to the heterogeneous nature of the included studies, it was not possible to perform a meta-analysis.

## Results

The initial search yielded 443 results. Based on our inclusion criteria, 17 were eligible for data extraction (Figure 1). A summary of the included studies is shown in Table 1. The studies were mainly published in 2014 or later, except for one study published in 2000. Nine studies were conducted in the US (5, 6, 11, 12, 16–20), four in Italy (21–24), one in France (7), one in Denmark (4), one in Canada (25), and one in Spain (26).

**TABLE 1:** Data extraction and results.

Authors Country	Study design Population description	Sample size (*N*)	Definition of emphysema	Description of study (%)	Prevalence of emphysema (%)	Limitations
Wenger et al. (16) USA	Cross-sectional study EXHALE	PLWH: 162 C: 128	Semi-quantitative scoring. Dichotomized: mild or greater >10%	IVDU: NA Current smoking: 59/52 cART: 70 Undetected. viral repl. *: 70 Mean age: 54 Male participants: 95	PLWH: 27 C: 15	Semi-quantitative scoring Limited generalizability
Besutti et al. (21) Italy	Cross sectional study Modena	PLWH: 159 C: 75	Semi-quantitative scoring. Dichotomized: mild or greater >10%	IVDU: 1.9 Current smoking: 0 cART: 100 Undetected. viral repl.: 98% Mean age: 55 Male participants: 87	PLWH: 18 C: 4	Semi–quantitative scoring Uninfected controls not fully matched.
Maitre et al. (7) France	Register study PMSI	PLWH: 10,067 C: 8,244,682	Not disclosed	IVDU: NA Current smoking: NA cART: NA Undetected. viral repl.: NA Mean age: NA Male participants: NA	PLWH: 2.6 C: 0.6	No definition of emphysema No description of the study
Ronit et al. (4) Denmark	Cross-sectional study COCOMO	PLWH: 742 C: 470	% LLA-950 threshold with cut-offs at 5% and 10%	IVDU: 1.6 Current smoking: 26/10 cART: 99 Undetected. viral repl: 95 Mean age: 55 Male participants: 86/82	PLWH: 5%: 22 10%: 5 C: 5%: 24 10%: 4	Uninfected controls were not fully matched.
Triplette et al. (5) USA	Cross-sectional study EXHALE	PLWH: 196 C: 165	Semi-quantitative scoring. Dichotomized: mild or greater >10%	IVDU: 32/18 Current smoking: 64/58 cART: NA Virally suppressed (<400 copies/ml): 83 Mean age: 55/53 Male participants: 98/89	PLWH: 31 C: 16	Semi-quantitative scoring Limited generalizability ART% not disclosed
Triplette et al. (6) USA	Cross-sectional study EXHALE	PLWH: 170 C: 153	Semi-quantitative scoring. Dichotomized: mild or greater >10%	IVDU: 31/15 Current smoking: 63/58 cART: 72 Undetected. viral repl: 65 Mean age: 55/52 Male participants: 98/88	PLWH: 31 C: 16	Semi-quantitative scoring Limited generalizability.
Triplette et al. (11) USA	Cross-sectional study EXHALE	PLWH: 190 Where 164 underwent CT	Semi-quantitative scoring. Dichotomized: mild or greater >10%	IVDU: 33 Current smoking: 63 cART: 71 Undetected. viral repl: 66 Mean age: 55 Male participants: 98	PLWH: 31	Semi-quantitative scoring Limited generalizability
Triplette et al. (12) USA	Cross-sectional study EXHALE	PLWH: 158 C: 133	Semi-quantitative scoring. Dichotomized: mild or greater >10%	IVDU: NA Current smoking: 62 cART: Undetected. viral repl: 67 Mean age: 53 Male participants: 94	PLWH: 33 C: 16	Semi-quantitative scoring Limited generalizability ART% not disclosed
Besutti et al. (22) Italy	Cross-sectional study Modena	PLWH: 1446	Semi-quantitative scoring. Total scores 0–4.	IVDU: NA Current smoking: 39 cART: 100 Undetected. viral repl: 94 Mean age: 48 Male participants: 71	PLWH: 35% 13 (>4) 22 (2–4)	Semi-quantitative scoring
Leader et al. (17) USA	Cross sectional study	PLWH: 510	LLA-950 threshold with cut-offs at 2,5% and 5%	IVDU: 24 Current smoking: 64 cART: 69 Virally suppressed (<400 copies/ml): 61 Mean age: 49 Male participants: 81	PLWH: >2,5%: 25.1 >5%: 9.2	Application of predefined threshold to assess emphysema. Lack of controls
Leung et al. (23) Italy	Cross sectional study Modena	PLWH: 345	Semi-quantitative scoring. Total scores 0–4.	IVDU: 25.5 Current smoking: 48 cART: 100 Undetected. viral repl: 77 Mean age: 49 Male participants: 90	PLWH: 41 (presence of emphysema)	Semi-quantitative scoring Lack of controls
Liu et al. (25) Canada	Cross sectional study	PLWH: 109 (underwent CT) 231	Semi-quantitative scoring. Total scores 0–4.	IVDU: 35 Current smoking: 55 cART: NA Undetected. viral repl: 70 Mean age: 50 Male participants: 91	PLWH: 13 (*n* = 30)	Semi–quantitative scoring Lack of CT-scanned controls ART% not disclosed
Attia et al. (18) USA	Cross-sectional study EXHALE	PLWH: 114 C: 89	Semi-quantitative scoring. Dichotomized: mild or greater >10%	IVDU: 32/10 Current smoking: 62/56 cART: 93 Virally suppressed (<400 copies/ml): 80 Mean age: 55/52 Male participants: 97/85	PLWH: 33 C: 17	Semi-quantitative scoring Limited generalizability
Guaraldi et al. (24) Italy	Cross-sectional study Modena	PLWH: 1446	Semi-quantitative scoring. Total scores 0–4. >1	IVDU: 28 Current smoking: 40 cART: 100 Undetected. viral repl: 94 Mean age: 48 Male participants: 71	PLWH: 41	Semi-quantitative scoring Lack of controls Not all participants underwent full lung scans.
Clausen et al. (19) USA	Cross sectional study	PLWH: 121	Semi-quantitative scoring. Total scores 0–4.	IVDU: 3.3 Current smoking: 80 cART: 85 Undetected. viral repl: NA Mean age: 45 Male participants: 68	PLWH: 26,4	Semi-quantitative scoring Lack of controls Self-reported demographics
Sampériz et al. (26) Spain	Cross sectional study	PLWH: 275	LLA-950 threshold with cut-offs at 1% Visual assessment	IVDU: 32 Current smoking: 62 cART: 96 Undetected. viral repl.: 92 Mean age: 49 Male participants: 79	PLWH: 11 Visual assessment: 38	Lack of controls Limited generalizability
Diaz et al. (20) USA	Cross-sectional study	PLWH: 114 C: 44	Semi-quantitative scoring. 0–10/lingua. >6 = presence of emphysema	IVDU: NA Current smoking: 60/56 cART: <10 Undetected. viral repl: NA Mean age: 34 Male participants: 90	PLWH: 15 C: 2	Semi-quantitative scoring Limited generalizability Low cART coverage

*Undetectable viral replication: <50 copies/ml. AIDS, acquired immunodeficiency syndrome; C, controls; cART, combination antiretroviral therapy; COCOMO, Copenhagen comorbidity in HIV Infection; COPD, Chronic obstructive pulmonary disease; CT, computed tomography scan; EXHALE, the examinations of HIV-associated lung emphysema study; HIV, human immunodeficiency virus; IVDU, intravenous drug users; LLA-950, % low attenuation area less than or equal to −950 Hounsfield units; Modena, the modena HIV metabolic clinic; NA, no information; PLWH, people living with HIV; USA, United States of America; VACS, veterans aging cohort study.

### Characteristics of study participants

Due to the highly heterogeneous characteristics of the participants in the included studies, a brief presentation of the included cohorts will follow. An overview of the study participants can be found in Table 1. An extended version can be found in Supplementary Table 1.

### The examinations of human immunodeficiency virus-associated lung emphysema study

In six studies, the participants were enrolled in the EXHALE study, which was a sub-study of the US Veterans Aging Cohort (VACS) (5, 6, 12, 16, 18, 27). All participants in EXHALE were former soldiers/veterans, and the study included both PLWH and controls. The population was predominantly male, >50 years, and of African-American ethnicity. There was a cART coverage of 70–93%, 59–64% were smokers, and 31–33% had a history of intravenous drug use (IVDU). In four of the studies 62–70% of participants had undetectable viral replication (6, 12, 16, 27). The last two reported viral suppression <400 ml/copies in 80 and 83% of participants, respectively (5, 18).

### The Modena human immunodeficiency virus metabolic clinic

Four of the studies were from an outpatient clinic in Italy, the Modena HIV metabolic clinic (21–24). The enrolled participants had >18 months of cART exposure (cART coverage 100%) and were >18 years old. Between 77 and 98% had undetectable viral replication. Among the participants, 1.9–28% had a history of IVDU, and 0–48% had a smoking history. However, one study only included never-smoking PLWH (21).

### Copenhagen comorbidity in human immunodeficiency virus infection

One of the included studies was from the COCOMO study which aimed to examine the prevalence, pathogenesis, and incidence of non-AIDS comorbidities in a well-treated cohort of PLWH. Most participants were on cART (99%), and 95% had undetectable viral replication. Of the included PLWH, 26% had a history of smoking, and 1.6% had a history of IVDU.

### Programme de médicalisation de systèmes d́Information

Programme de médicalisation de systèmes d́Information (PMSI) is a French nationwide hospital discharge database. Maitre et al. utilized this in a register-based study, where they included all PLWH > 18 who had been hospitalized from 2007 to 2013 for more than 1 day. HIV-negative individuals hospitalized in 2010 were used as controls (7). Smoking status, history of IVDU, cART coverage, and viral replication was not reported.

### Others

In five of the studies participants were not part of a larger cohort: Liu et al. (25), Clausen et al. (19), Diaz et al. (20), Leader et al. (17) and Sampériz et al. (26). A description of these studies is presented in Table 1.

### Definition of emphysema

Most studies defined emphysema either semi-quantitatively or quantitatively. The PMSI cohort defined emphysema according to the International Statistical Classification of Diseases and Related Health Problems (ICD-10) codes (7).

### Semi-quantitative definition

Most studies applied a semi-quantitative definition of emphysema by visual assessment of a CT scan (5, 6, 11, 12, 16, 18–25). In the studies from the EXHALE cohort, the degree of emphysema was characterized by a thoracic radiologist. Participants were scored from 0 (no emphysema) to 5 (>75% emphysema), followed by a dichotomization: Trace or no emphysema (≤10%) and mild or greater emphysema (>10%) (5, 6, 12, 16, 18, 27). In the four studies from the Modena cohort, three radiologists gave scores from 0 to 6 to each lobe and divided emphysematous changes by severity: 0 (absence of emphysema) to >4 (severe emphysema). Finally, they dichotomized the findings and defined presence of emphysema as a score ≥1 (21–24). In Liu et al., emphysematous changes were assessed by two radiologists (25). They gave each lobe and the lingula a score from 0 (absence of emphysema) to 4 (76–100% emphysema). Finally, a total score was given by summation from 0 (absence of emphysema) to >4 (severe emphysema). In the study by Clausen et al., emphysema was visually reviewed by one radiologist and one pulmonologist (19). They determined the extent, distribution and type of emphysema. Scores ranged from 0 (<5%) to 4 (51–75%). They did not mention from which score emphysema was defined, when reporting prevalence. In the study by Diaz et al., the extent of emphysema was given a score from 0 to 10 (20). Two radiologists assessed the CT scans. Emphysema was defined as a total score ≥6.

### Quantitative definition

Three studies defined emphysema by densitometry using the low attenuation area −950 Hounsfield Units (% LAA-950 HU) method.% LAA-950 HU was defined as low attenuation area in the lung parenchyma of less than −950 HU. A cut-off in percentage was used to describe the proportion of the lung below this threshold (28). In the COCOMO study, two cut-offs were defined:% LAA-950 >10 and >5% (4). In the Leader et al. study two cut-offs were defined% LAA-950 with cut-offs at >2.5 and >5% (17). Finally, Sampériz et al. defined emphysema as% LAA-950 with a 1% threshold (26).

### Prevalence of emphysema in people living with HIV

The reported prevalence of emphysema in PLWH ranged from 2.6 to 41%. The studies including participants from EXHALE found a prevalence of emphysema of 27% (16), 31% (5, 6, 11), and 33% (12, 18). The cohorts from Modena, found a prevalence of emphysema of 18% (21), 35% (22, 24), and 41% (23). The prevalence in the study by Liu et al. was 13% (25). The study by Clausen et al. reported a prevalence of 26% (19). Diaz et al. found the prevalence of emphysema to be 15% (20). The COCOMO study found that 21 and 4.7% had emphysematous changes at the 5 and 10% cut-offs, respectively. The prevalence of emphysema in Leader et al. was 25 and 9.2% at the >2.5 and >5% cut-offs, respectively (17). Sampériz et al. found a prevalence of 11% (26). In PMSI, they found a prevalence of emphysema of 2.6% out of 10067 PLWH without any AIDS-defining events. In Figure 2, visual presentation of the prevalence of emphysema in all the included studies is presented. The weighted average prevalence of emphysema in PLWH was 23% (95% CI: 16–30).

### Prevalence of emphysema in people living with HIV and controls

The prevalence of emphysema in both PLWH and controls was reported in nine studies (Figure 2). In eight of the nine studies, the prevalence of emphysema was significantly higher in PLWH than in controls. The COCOMO study found that the prevalence of emphysema in PLWH was not statistically different from that in controls. In COCOMO the prevalence of emphysema was 21 and 24% (*p* = 0.23) in PLWH and controls at the 5% cut-off, while it was 4.7 and 4% (*p* = 0.68) at the 10% cut-off (4). Studies from the EXHALE cohort found the prevalence of emphysema to be 27, 31, and 33% in PLWH, whereas the prevalence of emphysema in controls was 15% (16) (*p* < 0.05), 16% (5, 6) (*p* = 0.003), and 17% (18) (*p* = 0.01) respectively. The only study from the Modena cohort, including controls, was the study by Besutti et al., wherein all participants were never smokers. The prevalence of emphysema was determined to be 18% in PLWH and 4% in controls (*p* < 0.01) (21). Diaz et al. reported the prevalence of emphysema to be 15% in PLWH and 2% in controls (*p* = 0.025). Finally, the PMSI cohort found a 2.6% prevalence of emphysema in PLWH and 0.6% in controls (7). Further, of the seven studies including controls, only three reported the proportion of males in the control group, and it was comparable in PLWH and in the controls (4–6). In studies including both PLWH and controls the weighted average prevalence were 22% (95% CI: 10–33) and 9.7% (95% CI: 2.3–17), respectively (*p* = 0.052).

### Sensitivity analysis

In four studies that were published after 2016, the weighted average prevalence was 20% (95% CI: 3.2–38) and 9.8% (95% CI: 0.0–20) in PLWH and controls, respectively (*p* = 0.18).

### Risk of bias

**FIGURE 3:**
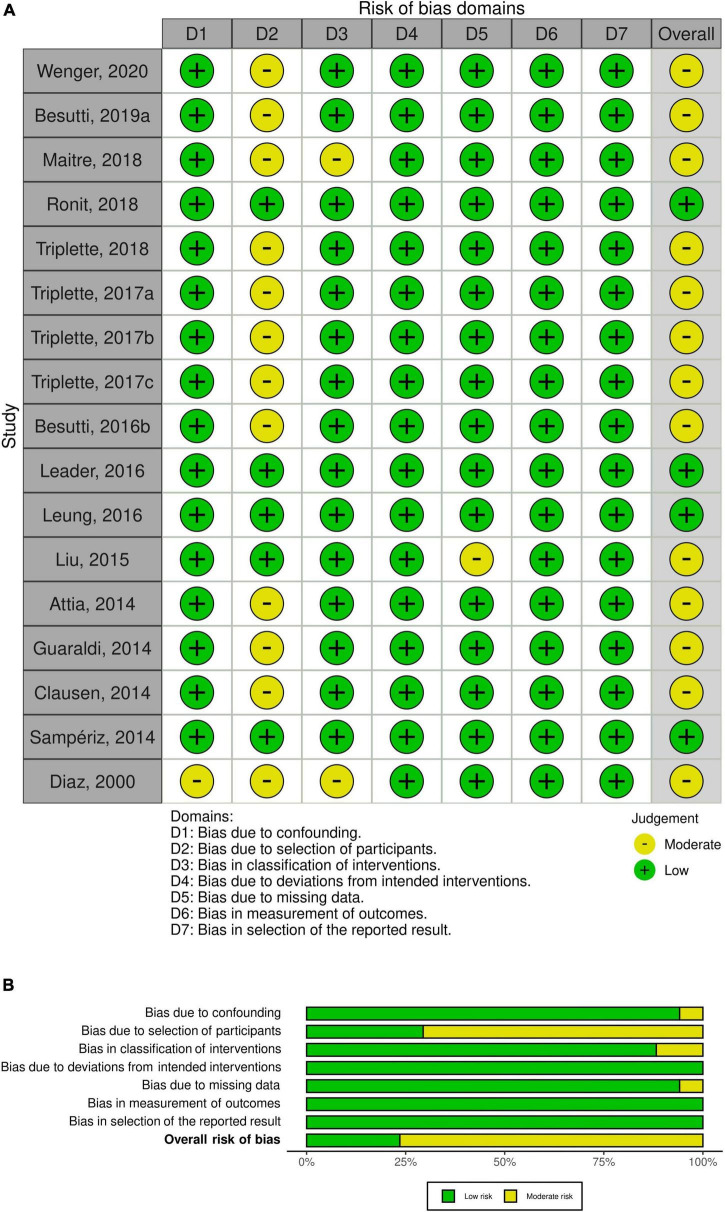
Risk of bias for the included studies. **(A)** Traffic light plots; **(B)** weighted bar plots for non–randomized clinical trial. The overall risk of bias was low in 4 of 17 studies and moderate in 13 studies.

The overall risk of bias was low for four out of seventeen studies. Most of the studies had moderate overall risk of bias mainly due to bias in selection of the participants. Figures 3A,B show a visual representation of bias assessment.

## Discussion

This systematic review aimed to investigate the prevalence of emphysema in PLWH and to compare the prevalence between PLWH and controls in the current cART era. The majority of the participants in the included studies were middle-aged smoking men receiving ART, and a considerable proportion had a history of intravenous or inhalational drug use. The weighted average prevalence of emphysema in PLWH was 23%.

The prevalence of emphysema was more than 10% in studies that determined emphysema semi-quantitatively using visual assessment (5, 6, 11, 12, 16, 18–25), while the reported prevalence was lower in studies that used a quantitative assessment method (4, 17, 26). Previous studies reported that there is merely a moderate agreement between semi-quantitative and quantitative assessments and the presence of emphysema (29). The semi-qualitative assessment, which radiologists often use, is experience-dependent and has inter-individual variations with risk of overestimation (30). The quantitative assessment is highly reproducible, can report a lower% LAA-950, and have a better correlation with microscopic and macroscopic emphysema than semi-quantitative methods (31–33). This might explain the differences in the prevalence of emphysema between the included studies, which use different methods.

Approximately half of the studies included controls, and a tendency toward a higher prevalence of emphysema was found in PLWH than in controls. However, although the same tendency was observed, the difference was not statistically significant when we only included four published studies after 2016. The prevalence of emphysema in never smoker PLWH and controls was only reported in one study and was almost fivefold higher in PLWH (21).

Smoking is a well-known risk factor for emphysema, and in studies that reported data on smoking, the prevalence of smoking was higher in PLWH than in controls (4–6, 16, 20). Although, other theories beyond tobacco smoking have been suggested to explain why the prevalence of emphysema might be higher in PLWH. Some of those are; a higher proportion of previous or recurrent respiratory infections (34, 35), presence of chronic inflammation (36), and also long-term exposure to cART (37). In the EXHALE cohort, the authors proposed that a CD4 nadir <200 cells/μl was an independent risk factor for emphysema, indicating that an immunocompromised state can predispose PLWH to develop emphysema (18). Moreover, the EXHALE proposed that a low CD4/CD8 ratio can be a risk factor for emphysema (27). Furthermore, it has been shown in a study from Denmark that a low nadir CD4 is associated with dynamic measures of pulmonary function (38). It is worth mentioning that a low CD4/CD8 ratio is a marker of the enduring immune activation in PLWH and residual inflammation. Even though, there are studies that did not find an association between emphysema and HIV-related factors, such as viral load and CD4-cell count (19, 23).

Our review has potential limitations; we only included English literature, while potentially relevant material may have been overlooked in other languages. Different standards for defining emphysema were used since no consensus gold standard exists, which could have led to difficulties in comparing results and both under- and overestimating emphysema. Most of the included studies had a risk of bias due to the selection of patients. Therefore, it may imply that the relatively high prevalence found in PLWH is only applicable to certain groups such as IVDUs. Finally, only one of the studies reported that the prevalence of emphysema did not differ between PLWH and controls; hence there is a risk of publication bias because of unpublished negative results.

## Conclusion

To conclude, the weighted average prevalence of emphysema in PLWH was 23%, and a tendency to higher prevalence of emphysema was found in PLWH than in controls in the current cART era. However, the definition of emphysema varied largely. Thus, to gain a clear overview, further studies with well-designed cohorts of PLWH and compared with controls are warranted.

## Data availability statement

The original contributions presented in this study are included in the article/Supplementary material, further inquiries can be directed to the corresponding author.

## Author contributions

HR, RT, and SN designed the study. HR and OR did the search. HR, RT, and OR screened manuscripts, determined risk of bias and extracted data, and wrote the first draft of the manuscript. SN and J-UJ commented and revised the manuscript. All authors read and approved the final version of the manuscript.
