# Supplementary material for: Prevalence of emphysema in people living with human immunodeficiency virus in the current combined antiretroviral therapy era: A systematic review
Source: Front Med (Lausanne). 2022 Sep 21;9:897773. doi: 10.3389/fmed.2022.897773 (PMC9532512; doi:10.3389/fmed.2022.897773)
Supplement: Supplementary file 1 [file Table_1.docx]

S1: Data extraction and results

| Authors | Country | Study design | Sample size (N) | Population description | Inclusion criteria | Definition of emphysema | Description of study (%) | Prevalence of emphysema (%) | Limitations |  |
| --- | --- | --- | --- | --- | --- | --- | --- | --- | --- | --- |
| Wenger et al. (2021) (16) | USA | Cross-sectional study | PLWH: 162  C: 128 | EXHALE | Included:  - Participants of VACS  - Matched on smoking history  Excluded:  - Self-reported drug abuse  - Incomplete smoking data | Semi-quantitative scoring. Dichotomized: mild or greater >10% | IVDU: NA  Current smoking: 59/52  cART: 70  Undetectable viral replication*: 70  Mean age: 54  Male participants: 95 | PLWH: 27  C: 15 | Semi-quantitative scoring  Limited generalizability |  |
| Besutti et al. (2019) (21) | Italy | Cross sectional study | PLWH: 159  C: 75 | Modena | - Documented HIV-infection  - 18 years or more  - cART exposure >18 months  - No history of smoking or opportunistic respiratory infections | Semi-quantitative scoring. Dichotomized: mild or greater >10% | IVDU: 1.9  Current smoking: 0  cART: 100  Undetectable viral replication: 98%  Mean age: 55  Male participants: 87 | PLWH: 18  C: 4 | Semi - quantitative scoring  Uninfected controls not fully matched. |  |
| Maitre et al. (2018) (7) | France | Register study | PLWH: 10, 067  C: 8,244,682 | PMSI | - PLWH hospitalized > 1 day between 2007-2013  - >18 years  - controls: HIV-negative persons hospitalized in 2010 | Not disclosed | IVDU: NA  Current smoking: NA  cART: NA  Undetectable viral replication: NA  Mean age: NA  Male participants: NA | PLWH: 2.6  C: 0.6 | No definition of emphysema  No description of the study |  |
| Ronit et al. (2018) (4) | Denmark | Cross-sectional study | PLWH: 742  C: 470 | COCOMO | COCOMO  - Only individuals over 40 chosen  Controls from CGPS:  - Only individuals over 40 CT-scanned  - Every 5^th^ women  - Every 5^th^ person over 70 | % LLA-950 threshold with cut-offs at 5% and 10% | IVDU: 1.6  Current smoking:26/10  cART: 99  Undetectable viral replication: 95  Mean age: 55  Male participants:86/82 | PLWH:  5%: 22  10%: 5  C:  5%: 24  10%: 4 | Uninfected controls were not fully matched. |  |
| Triplette et al. (2018) (5) | USA | Cross-sectional study | PLWH: 196  C: 165 | EXHALE | Included:  - Participants of VACS  - Matched on smoking history  Excluded:  - Chronic pulmonary diseases other than COPD or asthma. | Semi-quantitative scoring. Dichotomized: mild or greater >10% | IVDU:32/18  Current smoking: 64/58  cART: NA  Virally suppressed (<400 copies/ml): 83  Mean age: 55/53  Male participants: 98/89 | PLWH: 31  C: 16 | Semi-quantitative scoring  Limited generalizability  ART% not disclosed |  |
| Triplette et al. (2017) (6) | USA | Cross-sectional study | PLWH: 170  C: 153 | EXHALE | Included:  - Participants of VACS  - Matched on smoking history  Excluded:  - Chronic pulmonary diseases other than COPD or asthma. | Semi-quantitative scoring. Dichotomized: mild or greater >10% | IVDU: 31/15  Current smoking: 63/58  cART: 72  Undetectable viral replication: 65  Mean age: 55/52  Male participants: 98/88 | PLWH: 31  C: 16 | Semi-quantitative scoring  Limited generalizability. |  |
| Triplette et al. (2017) (11) | USA | Cross-sectional study | PLWH: 190  Where 164 underwent CT | EXHALE | Included:  - Participants of VACS  - Matched on smoking history  Excluded:  - Chronic pulmonary diseases other than COPD or asthma.  - Only PLWH with baseline measurements of CD4/CD8 | Semi-quantitative scoring. Dichotomized: mild or greater >10% | IVDU: 33  Current smoking: 63  cART: 71  Undetectable viral replication: 66  Mean age: 55  Male participants: 98 | PLWH: 31 | Semi-quantitative scoring  Limited generalizability |  |
| Triplette et al. (2017) (12) | USA | Cross-sectional study | PLWH: 158  C: 133 | EXHALE | Included:  - Participants of VACS  - Matched on smoking history  Excluded:  - Chronic pulmonary diseases other than COPD or asthma. | Semi-quantitative scoring. Dichotomized: mild or greater >10% | IVDU: NA  Current smoking: 62  cART:  Undetectable viral replication: 67  Mean age: 53  Male participants: 94 | PLWH: 33  C: 16 | Semi-quantitative scoring  Limited generalizability  ART% not disclosed |  |
| Besutti et al. (2016) (22) | Italy | Cross-sectional study | PLWH: 1446 | Modena | - Documented HIV-infection  - 18 years or more  - cARTexposure >18 months  - One CT scan for assessment of coronary artery calcium score  - No contradictions for CT scan: weight or pregnancy | Semi-quantitative scoring. Total scores 0-4. | IVDU: NA  Current smoking: 39  cART: 100  Undetectable viral replication: 94  Mean age: 48  Male participants: 71 | PLWH: 35%  13 (>4)  22 (2-4) | Semi-quantitative scoring |  |
| Leader et al. (2016) (17) | USA | Cross sectional study | PLWH: 510 |  | - >18 years  - Without acute respiratory illness | LLA-950 threshold with cut-offs at 2,5% and 5% | IVDU: 24  Current smoking: 64  cART: 69  Virally suppressed (<400 copies/ml): 61  Mean age: 49  Male participants: 81 | PLWH: >2,5%: 25.1  >5%: 9.2 | Application of predefined threshold to assess emphysema.  Lack of controls |  |
| Leung et al. (2016) (23) | Italy | Cross sectional study | PLWH: 345 | Modena | - Documented HIV-infection  - 18 years or more  - cART exposure >18 months | Semi-quantitative scoring. Total scores 0-4. | IVDU: 25.5  Current smoking: 48  cART: 100  Undetectable viral replication: 77  Mean age: 49  Male participants: 90 | PLWH: 41  (presence of emphysema) | Semi-quantitative scoring  Lack of controls |  |
| Liu et al. (2015) (25) | Canada | Cross sectional study | PLWH: 109 (underwent CT)  231 |  | - Documented HIV-infection  - 19 years or more | Semi-quantitative scoring. Total scores 0-4. | IVDU: 35  Current smoking: 55  cART: NA  Undetectable viral replication: 70  Mean age: 50  Male participants: 91 | PLWH: 13 (n=30) | Semi - quantitative scoring  Lack of CT-scanned controls  ART% not disclosed |  |
| Attia et al. (2014) (18) | USA | Cross-sectional study | PLWH: 114  C: 89 | EXHALE | Included:  - Participants of VACS  - Matched on smoking history | Semi-quantitative scoring. Dichotomized: mild or greater >10% | IVDU: 32/10  Current smoking: 62/56  cART: 93  Virally suppressed (<400 copies/ml): 80  Mean age: 55/52  Male participants: 97/85 | PLWH: 33  C: 17 | Semi-quantitative scoring  Limited generalizability |  |
| Guaraldi et al. (2014) (24) | Italy | Cross-sectional study | PLWH: 1446 | Modena | - Documented HIV-infection  - 18 years or more  - cART exposure >18 months | Semi-quantitative scoring. Total scores 0-4.  >1 | IVDU: 28  Current smoking:40  cART: 100  Undetectable viral replication: 94  Mean age: 48  Male participants:71 | PLWH: 41 | Semi-quantitative scoring  Lack of controls  Not all participants underwent full lung scans. |  |
| Clausen et al. (2014) (19) | USA | Cross sectional study | PLWH: 121 |  | - Documented HIV-infection  - 18 years or more  - Without acute respiratory illness  - No contraindications to CT – scan | Semi-quantitative scoring. Total scores 0-4. | IVDU:3.3  Current smoking: 80  cART: 85  Undetectable viral replication: NA  Mean age: 45  Male participants: 68 | PLWH: 26,4 | Semi-quantitative scoring  Lack of controls  Self-reported demographics |  |
| Sampériz et al. (2014) (26) | Spain | Cross sectional study | PLWH: 275 |  | - Clinical stability  - Age 40-69  - Signed informed consent  - Without chronic renal, heart or liver failure, opportunistic infection, previous pulmonary resection, treatment with steroids | LLA-950 threshold with cut-offs at 1%  Visual assessment | IVDU:32  Current smoking:62  cART: 96  Undetectable viral replication: 92  Mean age: 49  Male participants: 79 | PLWH: 11  Visual assessment: 38 | Lack of controls  Limited generalizability |  |
|  |  |  |  |  |  |  |  |  |  |  |
| Diaz et al. (2000) (28) | USA | Cross-sectional study | PLWH: 114  C: 44 |  | - Persons with history of Pneumocystis carinii pneumonia or other pulmonary complications of AIDS were excluded. | Semi-quantitative scoring. 0-10/lingua.  >6 = presence of emphysema | IVDU: NA  Current smoking: 60/56  cART: <10  Undetectable viral replication: NA  Mean age: 34  Male participants: 90 | PLWH: 15  C: 2 | Semi-quantitative scoring  Limited generalizability  Low cART coverage |  |

*Undetectable viral replication: <50 copies/ml

Abbreviations: **AIDS**, acquired immunodeficiency syndrome; **C**, controls; **cART**, combination antiretroviral therapy; **COCOMO**, Copenhagen comorbidity in HIV Infection; **COPD**, Chronic obstructive pulmonary disease; **CT**, computed tomography scan; **EXHALE**, The Examinations of HIV-Associated Lung Emphysema study; **HIV**, human immunodeficiency virus; **IVDU**, Intravenous drug users; **LLA-950**, % low attenuation area less than or equal to -950 Hounsfield units; **Modena**, The Modena HIV metabolic clinic; **NA**, no information; **PLWH**, people living with HIV; **USA**, United States of America; **VACS**, Veterans Aging Cohort Study
